# Cardiovascular disease and absenteeism in Dutch occupational health: a retrospective study in a regular working population

**DOI:** 10.1007/s12471-025-01989-6

**Published:** 2025-09-15

**Authors:** Ivo M van Dongen, Jobst Winter, Bart Aben, Gilbert W M Wijntjens, Ronak Delewi, Jan Siebers, Robin N Kok, Frederieke G Schaafsma

**Affiliations:** 1 De Nieuwe Arts, Occupational Health Service, Amsterdam, The Netherlands; 2 Department of Public and Occupational Health, Amsterdam UMC, Outpatient Clinic for Occupational Health Medicine, Amsterdam, The Netherlands; 3 Cardiologie Stichtse Vecht, Maarssen, The Netherlands; 4 Department of Research and Development, HumanTotalCare BV, Utrecht, The Netherlands; 5 HumanCapitalCare BV, Son, The Netherlands; 6 Department of Cardiology, Amsterdam Cardiovascular Sciences, Amsterdam UMC, University of Amsterdam, Amsterdam, The Netherlands

**Keywords:** Absenteeism duration, Sick leave, Absenteeism-related costs, Cardiovascular disease

## Abstract

**Background:**

There is very limited data available on the impact of cardiovascular disease (CVD) on absenteeism occurrence, absenteeism duration, and the associated rough cost-estimate for employers.

**Methods:**

We extracted routinely collected absenteeism data for the years 2019–2022 from a database maintained by two large, nationally operating occupational health services (*n* = 443,740). All diagnoses and included sickness cases were recorded > 6 weeks of absenteeism.

Descriptive statistics, including median values (IQR) and percentages, were calculated and compared using the Mann-Whitney U test and Pearson chi-square test. Subgroup comparisons were performed using the Kruskal-Wallis test. To analyse return-to-work over time, a Kaplan-Meier curve was constructed, and differences in return-to-work were assessed using the Log Rank (Mantel-Cox) test.

**Results:**

CVD is the primary cause of absenteeism in 3.2% of all absenteeism cases. The median duration of absenteeism following CVD was 119 working days (IQR 156; Q1–Q3 62.9–218.6) with a minimum rough cost-estimate to employers of € 37,000 per employee. The most frequently occurring CVD diagnoses were: acute myocardial infarction, cerebrovascular disease, cardiac arrhythmia, unspecified cardiovascular complaints and angina.

**Conclusions:**

CVD occurs frequently, results in prolonged absenteeism, and incurs high costs for employers. We strongly believe that CVD-related absenteeism should receive greater attention. Specifically, both in-hospital and outpatient treatments should place a stronger emphasis on work-related issues, including strategies for returning to work with or without tailored assignments in the workplace. This focus will help ensure that employees can sustainably return to work and continue to contribute to society.

**Supplementary Information:**

The online version of this article (10.1007/s12471-025-01989-6) contains supplementary material, which is available to authorized users.

## Introduction

The impact of cardiovascular disease (CVD) on employment capabilities and its associated costs for businesses and society has been poorly investigated. CVD is the foremost cause of mortality globally, and it’s associated with high morbidity [1]. Among all types of CVD, ischemic heart disease (IHD), cerebrovascular disease, and peripheral arterial disease (PAD) are the most prevalent.

There is a lack of current data on the precise incidence of CVD-related absenteeism and the additional costs incurred by companies or society due to absenteeism caused by CVD. Previous research has shown that CVD significantly contributes to increased workplace absenteeism [2–7]. Currently, the only available data are on IHD specifically. In 2002, the average absenteeism duration after IHD was 189 days in the Netherlands, and globally the average absenteeism duration after myocardial infarction varied from 38 days to 177 days [8]. In a Swedish publication, covering the years 2006–2008, the adjusted mean absenteeism days were 83.9 (95% CI 80.6–86.5) during the first year after an IHD event, and after stroke these were 179.5 (95% CI 172.4–186.8) days [5]. The occurrence of IHD led to a six-fold increase in absenteeism days, and the occurrence of stroke led to a 14-fold increase compared to the absenteeism days prior to the event [5].

**Fig. 1:**
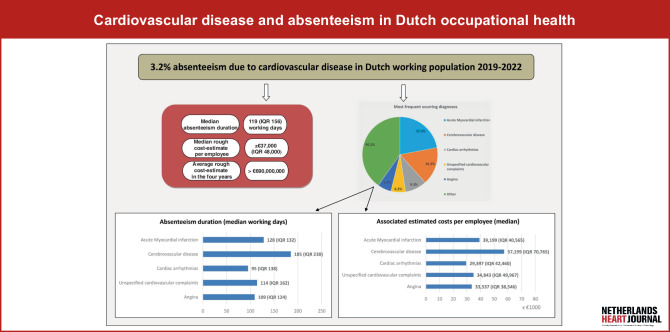
Infographic

Therefore, following an acute cardiac event, return-to-work (RTW) represents a vital patient-reported outcome (PROM) of interdisciplinary disease management [4]. Cardiac rehabilitation (CR) supports patients in recovering and RTW after acute coronary events, particularly through a multidisciplinary approach that includes medical, physical, psychosocial, and vocational support [9]. Despite this, many working-age patients do not successfully reintegrate into employment after CR, leading to reduced quality of life and societal costs [9]. Importantly, studies also show that RTW is more influenced by other PROMs—such as expectations, anxiety, and quality of life—than by clinical measures. Other factors, such as comorbidities, low education, and negative beliefs, hinder RTW while endurance training, higher education, and perceived work stress can facilitate RTW [9, 10]. With an aging workforce and rising retirement age, the burden of CVD on society is expected to grow, making effective vocational reintegration increasingly important (Fig. 1).

### Standard of care in absenteeism in the Netherlands

In the Netherlands, employees must report sickness to their employer when they are unable to work. If the absence is prolonged or expected to last more than a few weeks, the employee must consult an occupational health professional within the first 6 weeks. Employers are legally required to have a contract with such professionals to ensure access to occupational health services [11]. These professionals assess the cause of absenteeism, monitor recovery, and support RTW [11]. Employers must pay at least 70% of wages during the first two years of sick leave and may incur additional costs for absenteeism management and workplace adjustments. Wolvetang et al. have described the key legislation and procedures related to sick leave [11]; further background information on standard care in absenteeism is available as Electronic Supplementary Material (ESM).

### Study objectives

The present exploratory study aims to investigate the incidence of CVD-related absenteeism, the duration of absenteeism, and the associated costs for employers. Additionally, the study will identify the most frequently occurring CVD diagnoses to determine which specific CVD patients could benefit from more work-focused in-hospital and outpatient treatment.

## Methods

We extracted routinely collected absenteeism data from 2019–2022 from a database maintained by two large, nationally operating Dutch Occupational Health Services (OHSs; both are part of HumanTotalCare BV).

The Medical Research Ethics Committee of the Amsterdam UMC stated that the *Dutch Medical Research Involving Human Subjects Act* did not apply (reference number: 2024.0446).

### Study population

In the Netherlands, occupational health physicians (OHPs) record cardiac symptoms and diseases using the Dutch ‘Classification for Occupational and Social Insurances (CAS)’, based on ICD-10 [12]. This study included employees aged 16 or older who were reported sick due to a cardiovascular CAS code (C-class) between 2019 and 2022 [12]. Only cases with sick leave lasting at least 6 weeks (up to a maximum of 2 years, as per Dutch law) were included.

### Variables

Absenteeism duration, or time until RTW, was defined as the number of calendar days between the first day of sick leave and full return to work. Working days were calculated by multiplying total days by 5/7. To estimate employer costs, we used the friction method and hourly cost rates from Statistics Netherlands (CBS), which include wages, training, and social security contributions [11, 13–15]. In 2020, the cost per working day was € 38.11 × 8 h; in 2022 this was € 39.88 × 8 h [13–15]. We applied the 2020 rate to 2019–2021 and the 2022 rate to 2022. As data on overhead, exact wages, part-time work, or partial recovery were unavailable, cost estimates likely underestimate the true employer costs. All other variables were routinely collected during occupational healthcare and extracted anonymously from occupational healthcare medical records.

Further methodological details are provided in the ESM.

### Data analysis

Descriptive statistics were reported as mean (SD), median (IQR), or percentages. For group comparisons, we used the Mann-Whitney U test or Pearson chi-square test; subgroup differences were analysed with the Kruskal-Wallis test. Kaplan-Meier curves illustrated RTW over time for the five most common causes of absenteeism, with differences tested via the Log Rank (Mantel-Cox) test. Employees lost to follow-up—due to contract termination, retirement, or resignation—were censored and considered sick until that point. All *p*-values were two-sided, with significance set at *p* < 0.05. Analyses were performed using IBM SPSS Statistics version 28.0.1.1.

## Results

### Participants and disease occurrence

During the period 2019 to 2022, a total of 443,740 absenteeism cases were registered in the database. This database represents approximately 28% of the total population of employees on sick leave in the Netherlands during this timeframe [16]. In this database, the two most frequently occurring absenteeism diagnosis groups were mental illness (± 22%) and disease of the musculoskeletal system (± 17%). Over the four years, 14,209 absenteeism cases (3.2%) were recorded with a CAS code of interest for this study.

Table 1 presents the baseline characteristics of the included absenteeism cases. The study population is in general representative of the Dutch occupational population. However, there is a relative overrepresentation of employees in the manufacturing and construction sectors and an underrepresentation of the human health and social work activities sector in our study population.

**Table 1:** Baseline characteristics of employees included in the study (*n* = 14,209)

Variable	n	% of total
*Sex*		
Female	3562	25.1
Male	10,642	74.9
Unknown	5	0.0
*Age class (in years)*		
16–24	146	1.0
25–34	621	4.4
35–44	1437	10.1
45–54	4556	32.1
55+	7449	52.4
*Company employee count*		
0–500	10,059	70.8
500+	4094	28.8
Unknown	56	0.4
*Type of economic sector (top five)*		
Manufacturing	2650	18.7
Wholesale and retail trade; repair of motor vehicles and motorcycles	2270	16.0
Construction	1139	8.0
Transportation and storage	1025	7.2
Consultancy, research and other specialized business services	997	7.0
Other or unknown	6128	43.1
*Cardiovascular disease (highest to lowest prevalence)*		
Acute myocardial infarction	3128	22.0
Cerebrovascular disease	2309	16.3
Cardiac arrhythmia	1323	9.3
Unspecified cardiovascular disease	886	6.2
Angina	843	5.9
Unspecified ischemic cardiac disease	816	5.7
Unspecified cardiovascular complaints	758	5.3
Hypertension	684	4.8
Other cardiac disease	653	4.6
Cardiac dysrhythmia	635	4.5
Cardiac heart failure	440	3.1
High blood pressure	398	2.8
Chest pain	371	2.6
Pulmonary vascular disease	283	2.0
Congenital cardiovascular anomalies	166	1.2
Other trauma to vascular system	133	0.9
Cardiac conduction disorders	104	0.7
Previous myocardial infarction	96	0.7
Pulmonary heart failure	68	0.5
Traumatic intracranial bleeding	42	0.3
Hypotension	34	0.2
Other neoplasma of cardiovascular system	28	0.2
(Chronic) Rheumatic cardiac disease	11	0.1

The five most prevalent cardiovascular diseases were acute myocardial infarction (22.0%, *n* = 3128), cerebrovascular disease (16.3%, *n* = 2309), cardiac arrhythmia (9.3%, *n* = 1323), unspecified cardiovascular disease (6.2%, *n* = 886) and angina (5.9%, *n* = 843). An overview of the occurrence of all cardiovascular diseases is shown in Table 1.

### Absenteeism duration

**Fig. 2:**
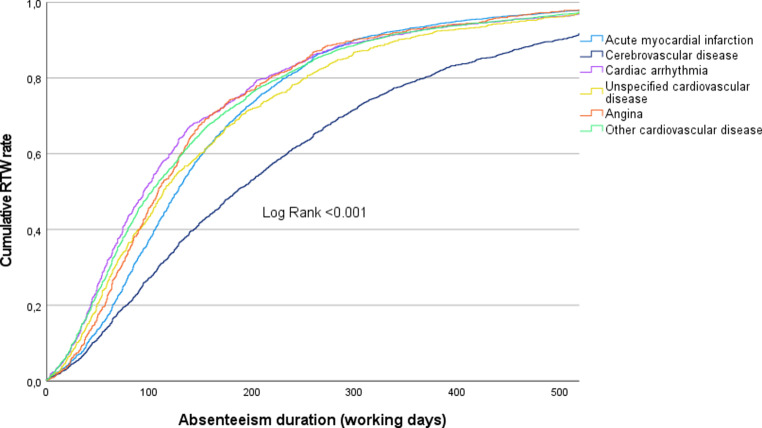
The figure displays the absenteeism duration and the cumulative RTW rate per diagnosis of the top five most occurring diagnoses in the overall population (*RTW* return-to-work)

The median duration of absenteeism due to cardiovascular disease in the period from 2019 to 2022 for this population was 119 working days (IQR 156; Q1–Q3 62.9–218.6). Figure 2 illustrates the absenteeism duration for the five most prevalent cardiovascular diseases with durations censored at 521 working days (= two calendar years).

Overall, 81.8% (*n* = 11,628) of the study population had fully returned to work within one year. Table 2 presents the average absenteeism duration and RTW rates categorized by time intervals (< 6 months, 6–12 months, and 12–18 months) for all CVD cases combined and for the five most prevalent diagnoses.

**Table 2:** Absenteeism duration and associated costs overall and per top five diagnoses

	All cardiovascular disease	Acute myocardial infarction	Cerebrovascular disease	Cardiac arrhythmias	Unspecified cardiovascular complaints	Angina	P value*
*Absenteeism duration (median working days*)	119 (IQR 156)	128 (IQR 132)	185 (IQR 230)	95 (IQR 138)	114 (IQR 162)	109 (IQR 124)	< 0.001
*Return to work (cumulative % of total)*							
< 6 months	54.2	51.5	36.8	64.2	55.5	59.7	< 0.001
6–12 months	81.8 (+27.6)	85.6 (+34.1)	65.1 (+28.3)	86.2 (+22)	81.3 (+25.8)	86.9 (+27.2)	< 0.001
12–18 months	92 (+10.2)	94.6 (+9.0)	82.8 (+17.7)	93.8 (+7.6)	92.5 (+11.2)	94.1 (+7.2)	< 0.001
*Associated rough cost-estimate per employee (median €)*	36,803 (IQR 48,341)	39,199 (IQR 40,565)	57,199 (IQR 70,765)	29,397 (IQR 42,460)	34,843 (IQR 49,967)	33,537 (IQR 38,546)	< 0.001

*Comparison between the top five diagnoses

### Absenteeism-related costs

The associated rough cost-estimate for CVD-related absenteeism in this population amounted to an excess of € 690 million over the four years. For every single employee with CVD, the median cost to employers mounts up to approximately € 37,000. Tab. 2 displays the average associated costs per case for all CVD cases combined and for the five most prevalent diagnoses.

## Discussion

In this exploratory study, we used standard-of-care data from two major occupational health services in the Netherlands to examine absenteeism due to cardiovascular disease (CVD) within a large cohort of the Dutch working population from 2019 to 2022. We observed a 3.2% incidence of absenteeism due to CVD, a median absenteeism duration of 119 working days, and a minimum rough cost-estimate for employers of around € 37,000 per employee. Of all CVD diagnoses, cerebrovascular disease was found to be associated with the longest absenteeism duration. For reference, in the Netherlands two frequently occurring causes of absenteeism or work disability are stress-related illness (average absenteeism duration 101 working days; average cost to the employer of € 19,151 per employee [11]) and low back pain (median absenteeism duration 95 days; mean cost to the employer of € 15,350 per employee [17]).

In our study acute myocardial infarction (AMI) and cerebrovascular disease were the two most frequently occurring diagnoses, with an average absenteeism duration of 128 days and 185 days, respectively. These absenteeism durations are quite similar to earlier findings, thus even though medical care has evolved over time, AMI and cerebrovascular disease are still associated with a relatively long absenteeism duration. This highlights the critical importance of focusing rehabilitation efforts on achieving return-to-work (RTW) after AMI and cerebrovascular disease. Effective rehabilitation can help employees resume work, enhance their quality of life, and reduce absenteeism costs.

RTW rates within 12 months after acute coronary syndrome (ACS) range from 67–93%, with an average delay of 2–3 months. Kruse et al. reported 79% RTW after coronary heart disease (CHD) admissions versus 93% after other diagnoses [2]. In our study, approximately 86% of employees returned to work within 6–12 months following AMI. RTW outcomes after CVD are influenced by medical (e.g., ventricular function, ischemia, rhythm stability), occupational (e.g., physical demands, shift work, commuting), and psychosocial factors (e.g., depression, self-perceived health, cognitive impairments) [18, 19]. A multinational review identified six barriers (job strain, anxiety, depression, comorbidity, older age, low education) and four facilitators (job control, work ability, good perceived health, high socioeconomic status) to RTW after CVD [19]. Non-fatal CHD is associated with increased absenteeism, presenteeism, delayed RTW, early labour force exit, and unemployment, challenges that likely extend to AMI survivors, who also face risks such as reduced job responsibilities, part-time work, lower income, and dismissal [5, 19].

Cardiac rehabilitation (CR) uptake in the Netherlands is suboptimal. A nationwide cohort study analysing 106,212 patients with CVD between 2013 and 2019 reported an initial increase in CR participation from 28% in 2013 to 41% in 2016, after which rates stabilized [20]. Previously, RTW rates following CR have shown to be 65% at six months and 67% at twelve months following CR, with predictors including younger age, non-manual work, self-employment, higher quality of life, and favourable exercise ECG outcomes [21]. Although one Danish study indicated a lower probability of RTW at three months following CR, it demonstrated a higher probability at nine and twelve months [22]. In the Netherlands, CR programs are multidisciplinary, comprising exercise training, cardiovascular risk management, psychological support, and occupational counselling, aligned with national and European guidelines. Regarding neurorehabilitation, 86% of patients with acquired brain injury returned to work immediately after a four-month vocational rehabilitation program, with 64% remaining employed at long-term follow-up [23]. Dutch neurorehabilitation similarly integrates physical, cognitive, psychological, and vocational interventions to enhance functional recovery and work reintegration. Interestingly, Yokota et al. demonstrated in a small study population that cardiac rehabilitation following standardized in-hospital rehabilitation improved exercise tolerance and functional strength in patients recovering from cerebrovascular stroke [24].

### Implications for clinical practice

Occupational health professionals play a key role in ensuring sustainable reintegration for employees recovering from significant health conditions such as CVD. Their responsibilities include helping employees regain optimal functioning, ideally returning to pre-CVD performance levels, or finding new roles when full recovery is not feasible. Effective reintegration requires enhanced collaboration among medical disciplines such as cardiologists, neurologists, rehabilitation physicians, and occupational health experts. An integrated approach focusing on rehabilitation participation and work-related factors can improve employees’ quality of life, reduce employer costs, and provide broader societal benefits, including higher tax revenues and reduced social security expenses [19]. Person-centred care and digital rehabilitation programs have been shown to improve return-to-work rates after ACS and assist professionals in maintaining progress while resuming work activities [19].

### Conclusions

This study demonstrates that cardiovascular disease (CVD) is a primary cause of absenteeism in at least 3.2% of cases within the Dutch labour force. CVD is associated with relatively prolonged absenteeism and incurs substantial costs for employers. Additionally, acute myocardial infarction, cerebrovascular disease, cardiac arrhythmias, unspecified cardiovascular disease, and angina are the most frequently occurring diagnoses among this working population.

We advocate for a greater focus on work-related aspects in both in-hospital and outpatient treatments for CVD. To achieve this, it is essential that in-hospital and outpatient medical specialists collaborate more effectively with occupational health professionals. Furthermore, there is a pressing need for the development of more work-focused guidelines to better integrate occupational health considerations into cardiovascular care.

## Limitations

This study, based on a day-to-day occupational healthcare database covering approximately 28% of Dutch employees with absenteeism, likely underestimates the true prevalence and impact of CVD due to delayed diagnosis registration, single-code recording, and overlap with mental health conditions. Extrapolation to the full workforce and to other countries requires caution given systemic differences. Additionally, absenteeism duration was censored for part of the cohort, presenteeism was not assessed, and the friction cost method used may underestimate the true economic burden, suggesting the actual impact of CVD is greater than reported. See the ESMfor a more detailed description of the limitations.

## Supplementary Information

Data extraction
