# Supplementary material for: Cardiovascular disease and absenteeism in Dutch occupational health: a retrospective study in a regular working population
Source: Neth Heart J. 2025 Sep 15;33(11):343–53. doi: 10.1007/s12471-025-01989-6 (PMC12550078; doi:10.1007/s12471-025-01989-6)
Supplement: Supplementary file 1 — Data extraction [file 12471_2025_1989_MOESM1_ESM.docx]

Electronic Supplementary Material *Data extraction*

The OHS cover small and medium-sized enterprises (SMEs; <250 employees) as well as large national and international enterprises (≥250 employees). Together, they provide occupational healthcare for over one million employees in the Netherlands and represent all business activities in the Dutch labour market. The data was fully anonymized by removing all personal employee identifiers before being made available to the researchers.

*Study population*

Certain CAS codes were excluded from the study due to their lack of direct or complete relevance to CVD (e.g., C103 Nosebleed, C104 Enlarged Lymph Node, C200 Lymph Node Metastasis).

The overall sick-leave duration was calculated for four distinct scenarios: (1) employees who reported sick before 2019 but had a notification of recovery filed after 1 January 2019; (2) employees who were on sick leave somewhere between 2019 and 2022 and had a notification of recovery filed later within this period; (3) employees who were on sick leave between 2021 and 2022 and had a notification of recovery filed within the same period; and (4) cases where a notification of recovery was not filed by the end of 2022, with absenteeism duration censored as of December 31, 2022.
In the database, the frequency of absenteeism occurrences is reported per employee. If an employee reports sick within the first four weeks after recovery, the initial absenteeism case is reopened. If a new period of absenteeism begins after these four weeks, a new absenteeism record is created for the same employee, regardless of whether the cause is the same or another cardiovascular illness. Consequently, the number of absenteeism cases in the database represents a cumulative total of all absenteeism events where CVD is the primary cause, rather than a count of distinct individuals with CVD-related absenteeism. In this database, 437 employees had more than one CVD-related absenteeism record (representing 6% of all cases).

*Standard of care in absenteeism in The Netherlands*

The absenteeism process in the Netherlands can result in significant costs for employers due to productivity losses, continued salary payments, and, in some cases, the need to hire temporary replacements. This system is designed to protect employees during periods of illness, ensuring they can fully concentrate on their recovery, compelling employers to support employees during their sickness, and encouraging employers to invest in healthy workplaces(11).

When return-to-work (either own or other work) is not possible due to medical reasons within the first two years, the employer may terminate the contract and is at this point no longer responsible for payment of salary. Consequently, the employee will have to apply for a disability benefit at the Dutch Social Security Agency(11).

*Data analysis*Descriptive statistics were calculated and presented as the mean (SD) for normally distributed data or the median (IQR) for non-normally distributed data, or as percentages of the total. For comparisons of descriptive statistics, the Mann-Whitney U test was employed, while the Pearson chi-square test was used for comparing percentages. Differences between subgroups were assessed using the Kruskal-Wallis test.

To illustrate RTW over time for the five most frequent causes of absenteeism Kaplan-Meier (KM) curves were used. Differences in RTW rates over time were compared using the Log Rank (Mantel-Cox) test. Employees lost to follow-up were censored and classified as being sick up to the day of loss to follow-up. Lost to follow-up occurs when a contract with the employer is terminated for any reason (i.e. temporary contract, reaching the retirement age, resignation). All the p-values were two-sided, and a p-value < 0.05 was considered to be statistically significant. All analyses were conducted using IBM SPSS Statistics version 28.0.1.1.

*Study limitations*

The data were derived from a database that only includes day-to-day occupational healthcare and does not encompass additional variables or broader health contexts. **The diagnosis** codes are recorded typically after six weeks of absenteeism. Consequently, the actual prevalence of CVD among employees might be somewhat higher than reported. Also, only one diagnosis code is registered and since CVD can often be associated with mental health conditions, the study may underrepresent the true extent of absenteeism due to CVD. It is possible that some cases are categorized under mental health issues, even when CVD is a significant contributing factor. Additionally, benign cardiac arrhythmias, such as atrial fibrillation, likely contribute significantly to physical absenteeism but may not be the sole reason for employees calling in sick.

**Furthermore,** the dataset represents approximately 28% of Dutch employees experiencing absenteeism. As such, extrapolations to the entire Dutch working population involve estimations. Translations to other countries should be done with some caution: especially the costs are higher in the Netherlands since there is a different system regarding absenteeism and occupational healthcare.

For one subset of employees, the absenteeism duration was censored at the end of 2022 for the purposes of this study. As a result, the study may underrepresent the actual absenteeism duration for this group within the study period. Importantly, presenteeism, particularly 'impaired work function' rather than 'sickness presenteeism', is believed to have a greater economic impact than absenteeism (25). However, accurately estimating the associated costs is extremely challenging, and this study was unable to assess presenteeism costs.

Also, methods used for cost calculations, such as the friction method, have their own pros and cons (15). For instance, the friction method may underestimate the true costs. Therefore, the actual financial impact of cardiovascular disease on employers is likely higher than the estimates provided in this study.

Lastly, the database used was an occupational health service database which does not include medical information such as the type of treatment given, medication used, whether an employee was enrolled in a rehabilitation programme or not nor did the database entail certain employee- or job-specific information such as the socioeconomic status, salary per employee, a potential part-time factor etcetera.
